# The effect of interleukin-6 signaling on severe malaria: A Mendelian randomization analysis

**DOI:** 10.1016/j.ijid.2023.02.008

**Published:** 2023-04

**Authors:** Fergus Hamilton, Ruth E Mitchell, Andrei Constantinescu, David Hughes, Aubrey Cunnington, Peter Ghazal, Nicholas J. Timpson

**Affiliations:** 1 MRC Integrative Epidemiology Unit, University of Bristol, Bristol, UK; 2 Infection Sciences, North Bristol NHS Trust, Bristol, UK; 3 Department of Infectious Disease, Faculty of Medicine, Imperial College London, London, UK; 4 Centre for Paediatrics and Child Health, Imperial College London, London, UK; 5 System Immunity Research Institute, Division of Infection and Immunity, Cardiff University, Cardiff, UK

**Keywords:** Malaria, IL-6, IL6R, Severe malaria, Cytokines

## Abstract

**Objectives:**

Severe malaria remains a deadly disease for many young children in low- and middle-income countries. Levels of interleukin (IL)-6 have been shown to identify cases of severe malaria and associate with severity, but it is unknown if this association is causal.

**Methods:**

A single nucleotide polymorphism (SNP; rs2228145) in the IL-6 receptor was chosen as a genetic variant that is known to alter IL-6 signaling. We tested this, then took this forward as an instrument to perform Mendelian randomization (MR) in MalariaGEN, a large cohort study of patients with severe malaria at 11 worldwide sites.

**Results:**

In MR analyses using rs2228145, we did not identify an effect of decreased IL-6 signaling on severe malaria (odds ratio 1.14, 95% confidence interval 0.56-2.34, *P* = 0.713). The estimates of the association with any severe malaria subphenotype were similarly null, although with some imprecision. Further analyses using other MR approaches had similar results.

**Conclusion:**

These analyses do not support a causal role for IL-6 signaling in the development of severe malaria. This result suggests IL-6 may not be causal for severe outcomes in malaria, and that therapeutic manipulation of IL-6 is unlikely to be a suitable treatment for severe malaria.

## Highlights

• Animal data suggest interleukin-6 (IL-6) signaling has a role in malaria.
• Genetic analyses have identified benefit of IL-6 inhibition in other infections.
• In malaria, the genetic analyses do not support therapeutic IL-6 inhibition.

## Introduction

Interleukin (IL)-6 is a critical cytokine in the innate immune response [1]. It is highly pleotropic, with major roles in inducing C-reactive protein (CRP) production in hepatocytes, the induction of other acute phase reactants (*e.g*., fibrinogen), and in the clusters of differentiation 4 cells [1]. It acts by binding to its IL-6 receptor (IL6R), either on a cell membrane (“classical” IL-6 signaling) or in solution (“trans-signaling”), where the soluble form of gp130 bufferS the response [2]. The IL-6-IL6R complex subsequently associates with glycoprotein 130 kDa (gp130) on the cell surface to activate a wide range of cellular processes (*e.g*., JAK-STAT activation) [2].

Although the systematic reviews of observational studies in adults and children have identified that increased IL-6 is associated with mortality, the exact role of IL-6 in pathogenesis of severe malaria remains unresolved, and it is unclear whether high levels of IL-6 are a cause or consequence of severe malaria [3]. In one large study, IL-6 levels in serum were significantly lower in Malian children with hyperparasitemia than those with lower levels of parasitemia, suggesting that IL-6 might be important for control of parasitemia [4]. However, the same study found higher levels of IL-6 in children with cerebral malaria than in children with noncerebral severe malaria, a finding replicated in a subsequent study in Malawi [5]. An experimental study in mice demonstrated that genetic IL-6Radeficient mice experienced reduced lethality from virulent *Plasmodium chabaudi* infection without obvious changes in parasitemia. The restoration of IL-6 “trans-signaling” by administration of soluble IL-6Ra resulted in lethality in the IL-6Ra-deficient mice, whereas the neutralization of trans-signaling by administration of soluble gp130 increased the survival in IL-6Ra-sufficient mice, providing convincing evidence for a pathogenic role of IL-6 by trans-signaling in this model [6] However, older studies in different mouse models, including the *P. berghei* ANKA experimental cerebral malaria model, found no evidence for a causal role of IL-6 in the pathogenesis of severe malaria [7]. Thus, observational studies of human malaria and studies in mice have not proven a causal role for IL-6 in severe malaria in humans, and alternative approaches are needed to address this question.

In contrast, in other severe infections, such as COVID-19, randomized trials have suggested that IL-6 is critical for pathogenesis, with evidence that blockade of IL-6 improves survival [8]. In the large, recent World Health Organization (WHO) meta-analysis, IL-6 receptor antagonists (*e.g*., tocilizumab) were associated with a 3-5% absolute decrease in mortality in critically ill patients with severe COVID-19 [8]. This trial evidence was predated by genetic studies using instrumental variable approaches, such as Mendelian randomization (MR) [9], that identified carriers of certain single nucleotide polymorphisms (SNPs) in the IL-6 receptor gene (*IL6R*) were relatively protected from severe COVID-19, a finding replicated in multiple independent cohorts [10], [11], [12], [13]. Recent similar studies also identified the same protective effect in sepsis, where variants in *IL6R* were again found to be protective against the development of sepsis, admission to critical care with sepsis, and death with sepsis [11].

In particular, the SNP rs2228145 (also known as Asp358Ala) in the gene *IL6R* has been identified as functional [14], with strong associations with biomarkers of IL-6 signaling (*e.g*., CRP, fibrinogen, plasma IL-6R levels [15]) and with a number of traits, such as cardiovascular disease [15]. Functional work has shown that this SNP causes proteolytic shedding of the IL-6 receptor, leading to higher plasma IL-6R but reduced downstream “classical” IL-6 pathway activity (*e.g*., reduced CRP, reduced fibrinogen) [15]. This SNP has therefore been widely used as a phenocopy of IL-6 blockade; although, given the intricacies of IL-6 signaling, that is likely an oversimplification [2,^11^,^16^,17].

Given the trial evidence of effectiveness in COVID-19 and supporting genetic evidence in sepsis, we aimed to investigate whether IL-6 downregulation might also lead to improved outcomes in severe malaria and whether this might represent a common target for severe infection. The major challenge in undertaking similar two sample MR analyses in malaria is the differing ancestries included in genetic studies of malaria compared with those used to identify exposures.

MR is a form of instrumental variable analysis whereby the genetic variation (SNPs) known to associate with an exposure (in this case, IL-6 signaling) is used to estimate the effect of that exposure on an outcome (in this case, malaria) and under certain assumptions, can provide causal estimates [9]. However, this relies on SNPs having the same effect on the exposure in the outcome dataset. As nearly all (>90%) of genome-wide association studies (GWAS) have been performed in people of European ancestry (EUR), whereas malaria genetic data are exclusively in non-European populations and linkage disequilibrium patterns and allele frequencies vary widely across genetic background; this poses a significant challenge to identifying SNPs that associate with an exposure and can be used in MR [18].

In this study, we aimed to first identify SNPs that alter IL-6 signaling across non-European populations. Subsequently, we then used these SNPs in the MR analyses to estimate the causal effect of IL-6 signaling on severe malaria.

## Methods

### Identification of exposures

We took two approaches to identifying exposures associated with IL-6 signaling to be used in MR. First, we used GWAS for CRP in UK Biobank, of which there are substantial (n ∼ 19,000) participants with non-EUR and identified variants that altered CRP but that were *cis* to *IL6R*. CRP is produced in hepatocytes in response to IL-6 [1] and is assumed to be a proxy for IL-6 signaling. This approach has been widely used in MR studies before [10,^11^,[15], [16], [17].

In European populations, the SNP rs2228145 (also known Asp358Ala) is known to alter levels of the soluble IL-6 receptor and CRP and has been shown to be functional in laboratory studies [14,19]. In previous studies, this SNP explains between 20% and 40% of the variance in IL6R levels and ∼1% of variance in CRP levels, whereas multiple large-scale genetic studies have implicated this SNP in a wide range of inflammatory and metabolic diseases [20,21]. Studies using admixture mapping in admixed African American populations have also identified that this SNP as a causal candidate [22].

We aimed to identify if rs2228145 was present and associated with CRP in populations outside Europe. First, we extracted SNPs within 500 kb up and downstream of the transcriptional start site of *IL6R* from previously performed GWAS of CRP across five non- EUR groups (as defined by Pan-UKBB: African ancestry [AFR], n = 6203; Central and South Asian ancestry; n = 8397; East Asian ancestry [EAS], n = 2564; admixed American ancestry [AMR], n = 937; and Middle Eastern ancestry, n = 1498) within UK Biobank, performed by the Pan-UKBB. All GWAS are available through the Integrative Epidemiology Unit (IEU) OpenGWAS website [23], with details on genetic preprocessing, quality control, definition of continental ancestry group, and GWAS methodology on the Pan-UKBB website [24]. Briefly, CRP was inverse-rank normal transformed and GWAS was performed in each ancestry separately using SAIGE, a linear-mixed-model approach [25].

Subsequently, we meta-analyzed these five GWAS using METAL [26] to identify any heterogeneity and to identify if rs2228145 or any other SNPs had reliable associations in a transancestry analysis. After the meta-analysis, all SNPs that had a genome-wide association (*P* <5 × 10^−8^) were linkage dysequilibrium (LD) clumped (r^2^ <0.01) using a 1000 Genomes African reference panel to identify if there was more than one independent signal at this locus [27]. The identified SNPs were taken forward into the MR analyses.

To increase the precision of our effect estimates and because the effect estimates were similar across ancestries, we then included the much larger European ancestry GWAS performed by the Pan-UKBB (EUR, n = 400,094) in our meta-analysis to generate the effect estimates and standard errors [24].

### Secondary exposures

As a secondary exposure and attempt to interrogate other aspects of the IL-6 axis, we used a recent GWAS of plasma protein levels performed in an AFR population (Atherosclerosis Risk in Communities Study [ARIC] study, n ∼ 1500) [20]. We identified three proteins associated with IL-6 function (IL-6 itself and IL6R and gp130, which binds to IL6R to perform signaling [1]) and identified variants within 500 kb of the transcription start site of each protein that had a genome-wide significant association with the protein (*P* <5 × 10^−8^) and then performed LD clumping (r^2^ <0.01) with the 1000 Genomes AFR LD reference panel using the TwoSampleMR package [23]. These variants were then weighted by their association with the protein and taken forward for MR.

### Outcomes

For the measurement of SNP-outcome associations, we used the large, geographically diverse MalariaGEN study, which includes 11 populations, nine of which are in Africa [28]. The detailed inclusion criteria are with the original study, but briefly, this study recruited cases of severe malaria using the WHO definition with population control and performed a GWAS in each ancestry of severe malaria case status, followed by a meta-analysis across all sites [28]. Severe malaria was diagnosed by the WHO criteria [29]. They classified severe malaria into cerebral malaria, severe malarial anemia, and “other” severe malaria. They defined this as severe malaria with other malarial symptoms [28].

For our primary exposure (CRP), we used all MalariaGEN populations, but because our secondary exposures were all *cis-*protein quantitative trait loci (pQTLs) from AFR populations, we limited our study to the nine African populations.

The CRP associated SNP rs2228145 was directly genotyped in MalariaGEN using the Illumina Infium Omni 2.5M chip and has an INFO score of 1 across all study populations.

### MR and meta-analysis

MR is a form of instrumental variable analysis that, under certain assumptions, can provide the causal estimates of the effect of an exposure on an outcome. These assumptions are that the genetic instruments that are associated with the risk factor of interest were independent of potential confounders and could only affect the outcome through the risk factor and not through alternative pathways (*i.e*., through pleiotropy) [9].

We performed MR using the rs2228145 SNP as an instrument (for our primary exposure, CRP) and used the SNP-CRP exposure from the cross-ancestry meta-analysis to generate exposure weights. The MR estimates were generated using the Wald ratio or through inverse variance weighting, when there was more than one SNP for each pQTL for our secondary exposures. The MR estimates were then meta-analyzed across each study site in an inverse variance-weighted meta-analysis.

The MR estimates from our primary exposure (CRP) analysis are in rank-inverse normal transformed units of change in CRP. MR was performed for each of the three severe malaria subtypes (severe malarial anemia, cerebral malaria, and other severe malaria), with the meta-analysis across populations as described previously. For the sensitivity analyses, for our *cis*-pQTL exposure, we also performed meta-analyses using MR Egger and weighted median approaches. These approaches rely on different assumptions and are alternative meta-analytic strategies for calculating the summary MR estimates [9].

The analyses were performed using the TwoSampleMR package [23] and R version 4.0.4 (R Foundation for Statistical Computing, Vienna).

### Guidelines

This study is reported in line with the STROBE-MR guidance, which is available as a supplement (Supplement S1) [30].

### Data availability

This study was performed using publicly available data. MalariaGEN summary statistics are available at the MalariaGEN website [31], whereas the Pan-UKBB GWAS are available through the Pan-UKBB website [24] and through the IEU OpenGWAS website [23].

## Results

### Identification and assessment of variants at IL-6 receptor and association with CRP across ancestries

Across the six ancestry groups tested (European, Middle Eastern, African, Central South Asian, Admixed American, and East Asian), rs2228145 was consistently associated with CRP levels, with little evidence of heterogeneity of effect (Table 1, *P*-value for heterogeneity 0.67). The summary beta was -0.11 (SE = 0.012, *P* = 3.4 × 10^−21^) for each additional C allele, excluding the European ancestry subpopulation and -0.11 (SE = 0.003, *P* = 7.55 × 10^−255^), including the much larger European ancestry subpopulation.

**Table 1:** Effect of the rs2228145 allele on CRP across multiple continental ancestries in each of the Pan-UKB continental ancestry groups. CRP was inverse-rank normal transformed and so, betas reflect a one-SD change in inverse-rank normal transformed CRP. The MAF is from UK Biobank.

Ancestry	Beta	SE	*P*-value	MAF	n
*Individual ancestries:*					
African (AFR)	-0.111	0.03154	4.3×10^−4^	0.098	6203
Admixed American (AMR)	-0.134	0.04838	5.3×10^−3^	0.450	937
Central South Asian (CSA)	-0.101	0.01565	8.7×10^−11^	0.311	8397
East Asian (EAS)	-0.144	0.02910	7.1×10^−7^	0.342	2564
European (EUR)	-0.106	0.00260	1.4×10^−320^	0.410	400,094
Middle Eastern (MID)	-0.087	0.03549	1.35×10^−02^	0.358	1498
*Meta-analysis*					
Without European	-0.110	0.0116	3.37×10^−21^		19,599
With European	-0.106	0.0031	7.55×10^−320^		419,693

CRP, C-reactive protein; MAF, minor allele frequency.

The smallest beta was on the Middle Eastern ancestry population (β = -0.087) and the largest in the EAS (β = -0.144), with study-specific and meta-analyzed results available in Table 1.

**Figure 1:**
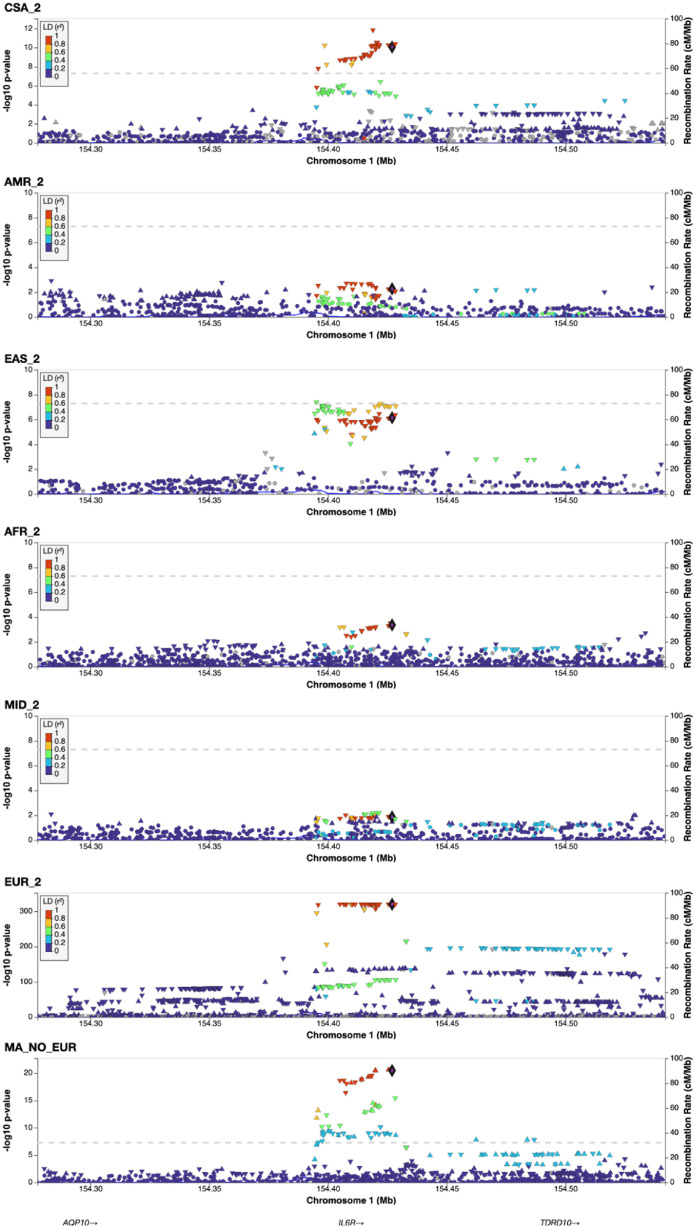
LocusZoom plots of the IL6R locus across each tested ancestry. LD values from the closest 1000 Genomes reference population. rs2228145 is the reference allele across all images (black dot). Definitions: AFR_2, African; AMR_2, Admixed American; CSA2, Central South Asian; EAS_2, East Asian; EUR_2, European, IL6R, interleukin-6 receptor; MA_NO_EUR, Meta-analysis of all studies except European; MID_2, Middle Eastern.

The minor allele frequency was similar in all populations outside of Africa (0.30-0.40) but was much lower in the AFR group (0.098). The locus plots of this region are available in Figure 1.

We did not identify any other independent (r^2^ <0.01) variants within 500 kb of the transcriptional start site of *IL6R* that had genome-wide significance for CRP at this in the meta-analyzed (excluding Europe) group. In summary, across all six continental ancestry groups, rs2228145 is associated with CRP (as a marker of IL-6 downregulation) and has an approximately similar effect size in all population, but the frequency of the C allele was lower in those of the AFR population.

### Identification of secondary exposures in the ARIC study

As secondary analyses, we aimed to generate secondary instruments for other aspects of the IL-6 pathway. From the ARIC study [20], we extracted GWAS for IL-6, IL6R, and gp130, and identified independent *cis*-pQTLs for each protein. We identified no *cis*-pQTLs for IL-6, three for the IL6R (including rs2228145), and one for gp130. The included SNPs are listed in Table S1.

### MR

**Figure 2:**
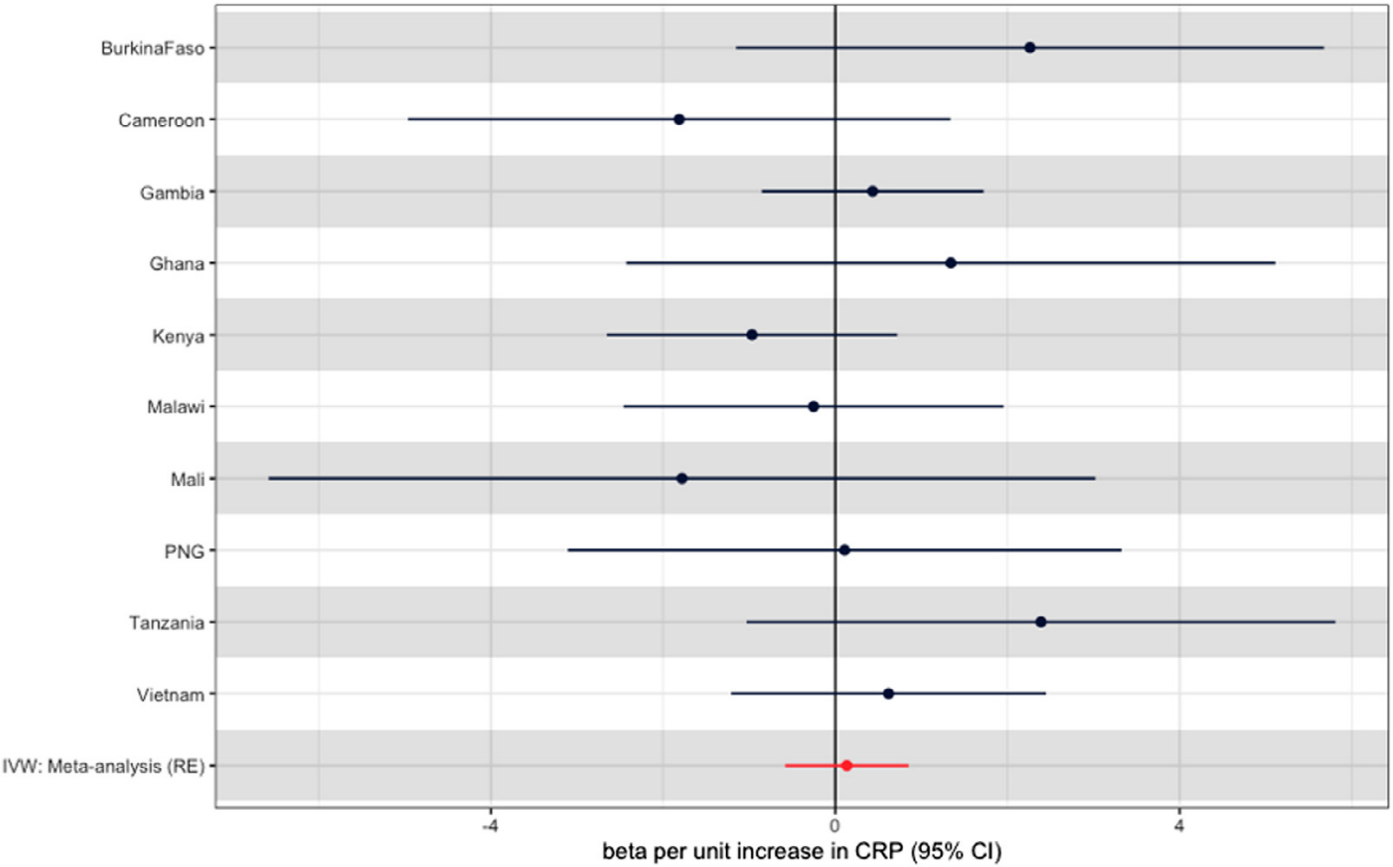
Mendelian randomization effect estimates for each population and the summary inverse variance-weighted beta for the rs2228145 single nucleotide polymorphism. Note, Nigeria not shown due to the presence of a single case of severe malaria (Table 2). Effect estimates generated by the Wald ratio. CI, confidence interval; CRP, C-reactive protein; IVW, inverse variance weighting.

For our primary exposure (IL-6 signaling as measured by CRP), we performed MR using the sole variant rs2228145 as an instrument for CRP. Across all populations, we saw little evidence of any effect of IL-6 signaling on severe malaria, with all estimates crossing the null but with a large degree of imprecision reflected by wide confidence intervals (CIs) across all estimates (Figure 2). In the meta-analysis, our summary result was close to the null, with an odds ratio (OR) of 1.21 (95% CI 0.51-2.88, *P* = 0.67) per each normalized unit increase in CRP. Figure 2 shows the results of this analysis, with each individual population and the summary random effects meta-analysis result, with number of cases at each site and study site effect estimates in Table 2. We did not identify evidence of study site-specific effects (I^2^ = 0, *P* for heterogeneity = 0.532).

**Table 2:** MR Effect estimates for each of the 11 populations, generated by a Wald ratio, and number of cases at each site. MR estimates are on the scale of a one-SD increase in inverse-rank normal transformed C-reactive protein.

Country	Odds ratio	*P*-value	Total number	Cases (% total)	Severe malarial anemia (% cases)	Cerebral malaria (% cases)
Burkina Faso	9.6 (0.32-291.82)	0.194	1327	733 (55.2%)	28 (3.8%)	94 (12.8%)
Cameroon	0.16 (0.01-3.81)	0.260	1277	592 (46.4%)	66 (11.1%)	32 (5.4%)
Gambia	1.54 (0.43-5.6)	0.509	5091	2487 (48.9%)	456 (18.3%)	780 (31.4%)
Ghana	3.83 (0.09-165.93)	0.485	716	396 (55.3%)	41 (10.4%)	31 (7.8%)
Kenya	0.38 (0.07-2.05)	0.261	3261	1646 (50.5%)	174 (10.6%)	690 (41.9%)
Malawi	0.78 (0.09-7.07)	0.823	2499	1182 (47.3%)	65 (5.5%)	642 (54.3%)
Mali	0.17 (0-20.51)	0.467	446	263 (59%)	81 (30.8%)	61 (23.2%)
Nigeria	0 (0-822.19)	0.295	131	109 (83.2%)	1 (0.9%)	28 (25.7%)
PNG	1.11 (0.04-27.77)	0.947	770	396 (51.4%)	115 (29%)	49 (12.4%)
Tanzania	10.91 (0.36-333.18)	0.171	807	409 (50.7%)	178 (43.5%)	31 (7.6%)
Vietnam	1.86 (0.3-11.57)	0.507	1264	718 (56.8%)	23 (3.2%)	154 (21.4%)
Inverse variance weighting: meta-analysis (random effects)	1.21 (0.51-2.88)	0.670	17,589	8,931	1,228	2,592

MR, Mendelian randomization.

**Figure 3:**
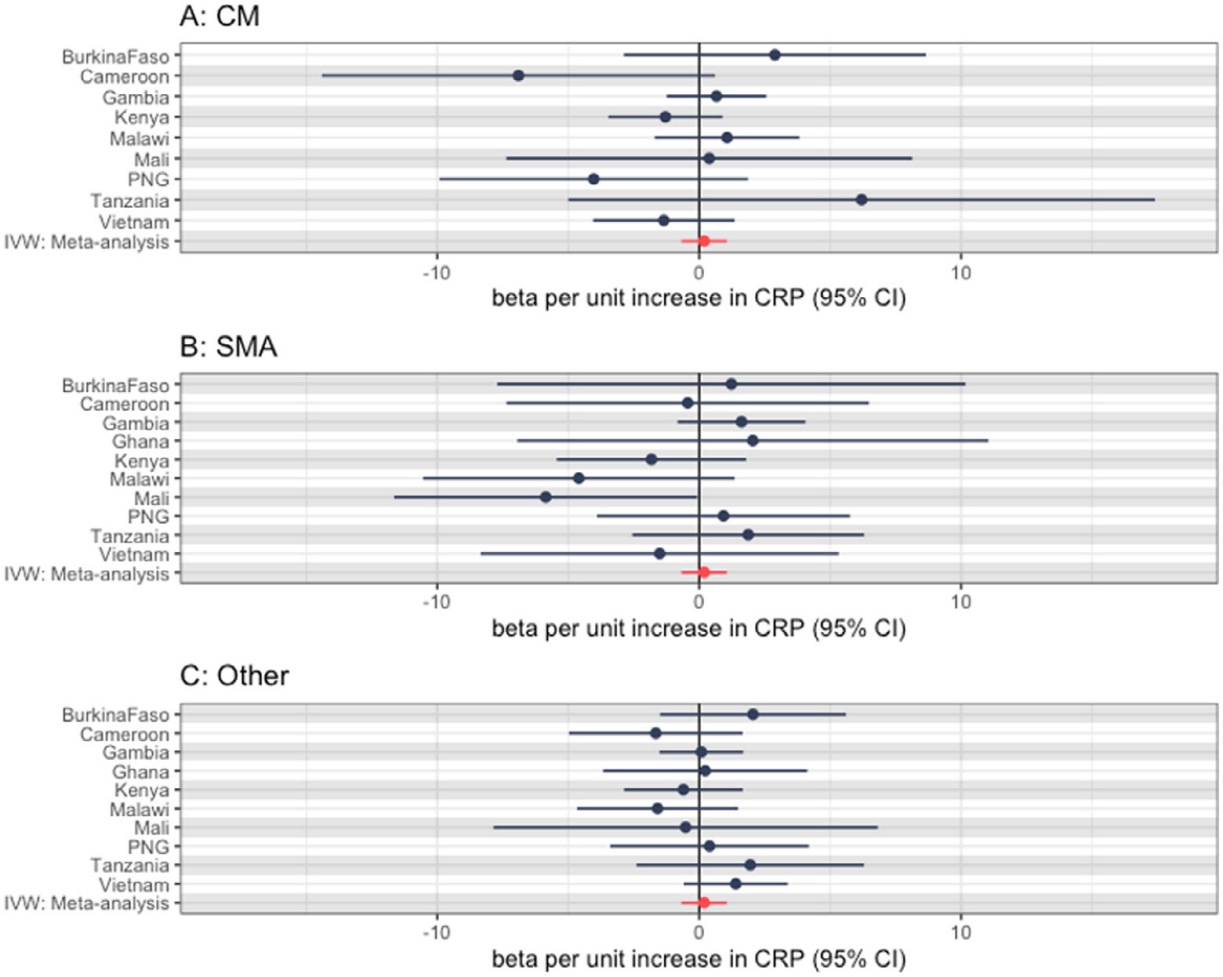
Mendelian randomization effect estimates (using the Wald ratio) for each severe malaria subtype using the rs2228145 single nucleotide polymorphism, with the summary IVW meta-analysis effect estimate also (a: CM, B: SMA, C: other). Note, Nigeria not shown again for all subtypes due to low case numbers, Ghana not shown for CM subtype only due to low case numbers. CI, confidence interval; CM, cerebral malaria; CRP, C-reactive protein; IVW, inverse variance weighting; SMA, severe malarial anemia.

We then went on to perform MR for three malaria subtypes. As with the main analysis, these results were largely null but were imprecise and did not preclude small effects. Figure 3 shows these effects for each subphenotype of severe malaria, with Table S2 showing the raw estimates.

### Secondary exposures

**Figure 4:**
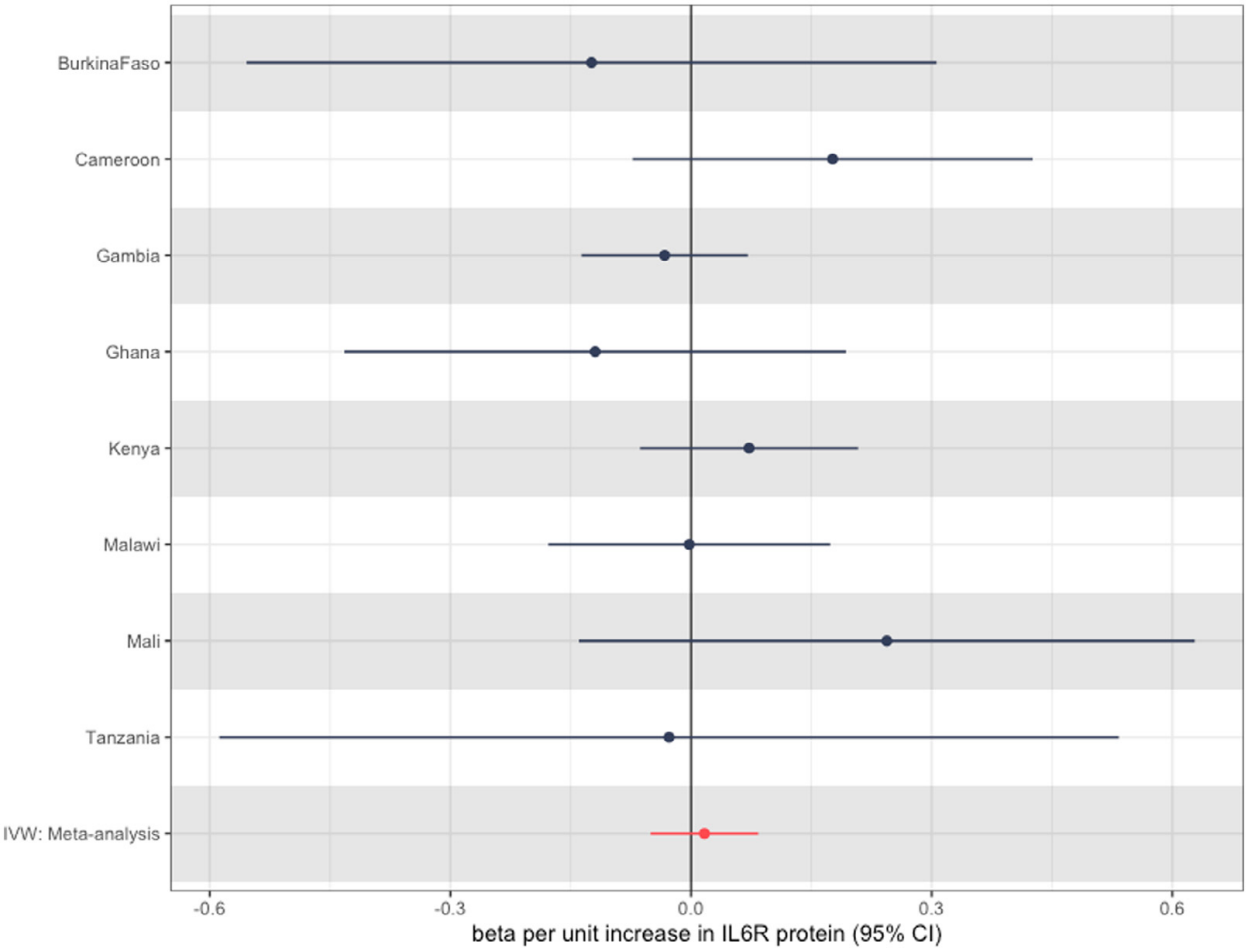
Inverse variance-weighted Mendelian randomization estimates for each study site for the association between *IL6R* protein levels and severe malaria case status. These are on the scale of an SD increase in inverse-rank normalized transformed IL6R protein levels. Note Nigeria again not shown due to low numbers of cases. CI, confidence interval; IL6R, interleukin-6 receptor; IVW, inverse variance weighting.

We then went on to perform MR using *cis*-pQTLs for *gp130* and *IL6R* generated from the ARIC study [20], which was undertaken in an African American population. At the *IL6R* locus, we identified three *cis*-pQTLs (one of which was rs2228145), enabling us to perform inverse variance-weighted meta-analysis and increase our power. The analyses yielded a summary MR estimate of an OR of 1.02 (95% CI 0.95-1.10) per each SD increase in inverse-rank normalized IL6R protein levels, with CIs for all study sites crossing the null (Figure 4). Alternative meta-analysis methods (MR Egger and weighted median) are reported in Table S3 but had very similar results.

**Figure 5:**
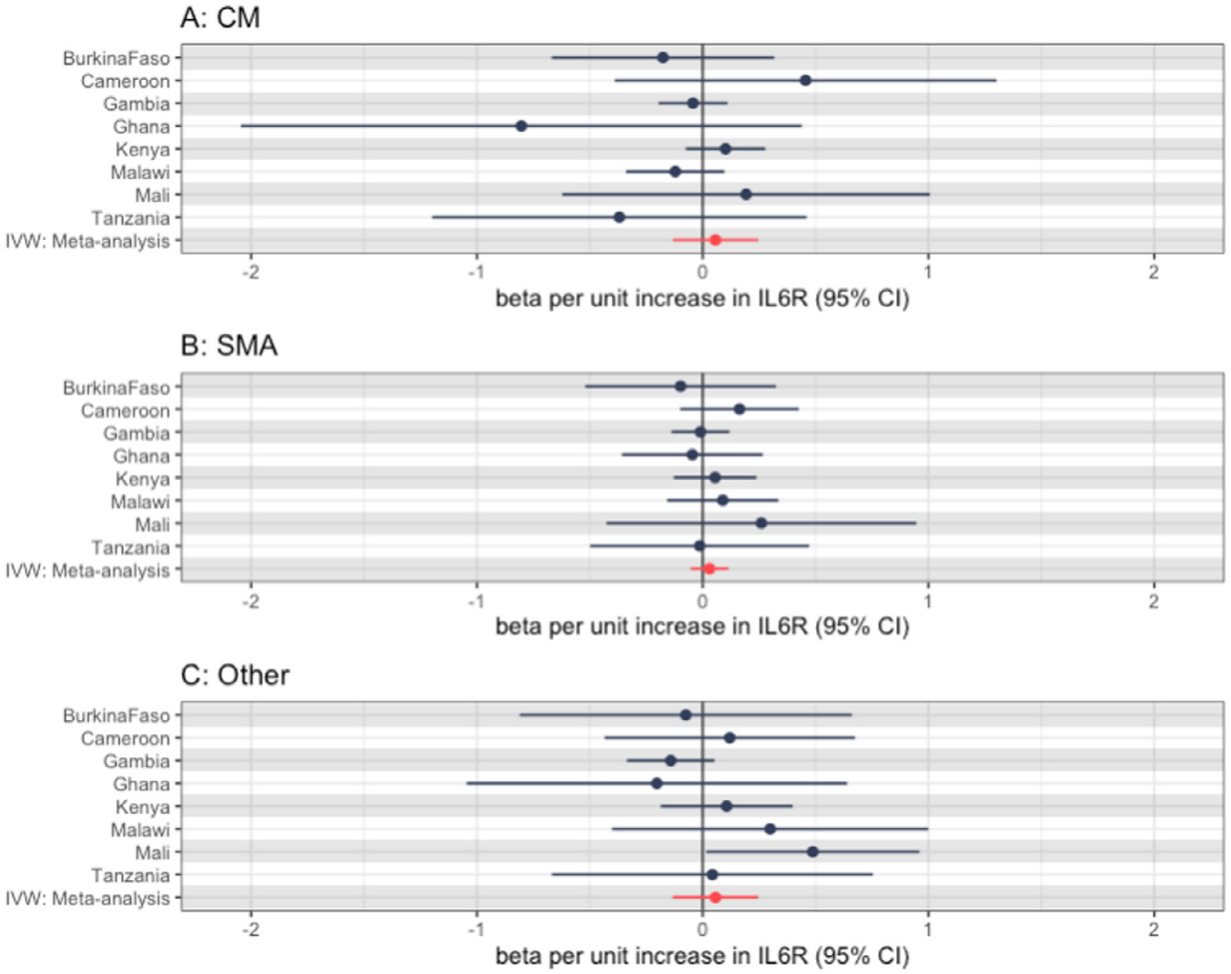
Mendelian randomization effect estimates (using the Wald ratio) for each severe malaria subtype (a: CM, b: SMA, c: Other). Note, Nigeria not shown again due to significant imprecision. CI, confidence interval; CM, cerebral malaria; IVW, inverse variance weighting; SMA, severe malarial anemia.

When looking at the subphenotypes of malaria, we saw a similar null result, with again a degree of imprecision due to low case numbers of each subtype at certain sites. Figure 5 shows this, with results shown in Table S4.

### gp130

*gp130* encodes for the protein gp130 (also known as IL6ST), which is the other subunit of the IL-6 receptor. We identified one *cis-*pQTL for this and performed MR using the Wald ratio to generate the estimate. Again, we identified a null effect, with a summary OR of 1.06 (0.88-1.28) for each SD increase in gp130 (Figure S1), with similar results for each malaria subtype (Figure **S2**). The results for the main analysis are shown in Table S5, with results for the subtype analyses in Table S6.

## Discussion

In this study, we investigated whether SNPs that were selected on the basis of interrupting IL6R and assumed to be a proxy for IL-6 signaling are associated with severe malaria case status.

Firstly, we showed that the rs2228145 variant in *IL6R*—a known splicing variant—associates with CRP levels (a marker of IL-6 signaling) across all tested ancestries in the UK Biobank and explains around 40-60% of the variance in IL6R levels in people of AFR [20] and therefore represents a good instrument to perform MR across non-European ancestries. We then took this forward in the MR analyses. We could not identify any effect of rs2228145 on severe malaria case status or any severe malaria subphenotype; although, the estimates remained imprecise. However, when subsequently using multiple *cis*-pQTLs for *IL6R*, which are known to alter downstream IL-6 signaling, we identified the same apparently null result, with more precision in the African populations included in the MalariaGEN [28]. In addition, the analyses undertaken did not identify any association with other proteins in the IL-6 signaling pathway.

This work leaves several questions about the role of IL-6 in severe malaria. A recent meta-analysis confirmed that IL-6 is prognostic for severe outcomes and differentiates uncomplicated from severe malaria [3]. However, our work suggests that, in contrast to COVID-19 (and perhaps bacterial sepsis), those with genetically proxied reduced IL-6 signaling do not have altered risk of severe disease [10,11]. IL-6 levels are raised (and associate with) a huge number of conditions, and so, it is plausible that IL-6 may represent a useful biomarker but one that is not causally related to severe malaria pathogenesis. Supporting this, the results of animal models of IL-6 manipulation in malaria have been inconsistent [6,7]. In summary, our work supports the hypothesis that increased IL-6 is not causal for severe malaria.

### Limitations

Like many MR studies, this study is limited by the available exposure and outcome data, and the assumptions of MR. Because all populations in MalariaGEN are outside Europe and there are few non-European large-scale GWAS of inflammatory biomarkers, we are limited to using exposure data from the non-European population of the UK Biobank (n ∼ 19,000) [32] and the recently published ARIC study (n ∼ 1500) [20]. As far as we are aware, there are no other available sources of potential data in non-European populations. For contrast, the recent GWAS of CRP in European ancestry populations included 557,000 people [33].

Because of this, for our main exposure, we were limited to using the well-understood rs2228145 SNP, which has been widely used for MR in European ancestry populations [11,^15^,34]. Although *in vitro* work has confirmed that this variant reduces cell surface expression of IL6R [14] and large-scale transcriptome studies in healthy patients [35] and those with infection [36] have shown this is a splicing variant, the use of a single variant reduces the statistical power of the study. This is compounded by the low minor allele frequency (∼10%) in AFR populations, reducing the power to identify small associations further. In order to overcome this, our secondary exposure used multiple *cis*-pQTLs for IL6R, a key IL-6 signaling molecule, and identified the same null result but with much greater precision, supporting the lack of effect at this locus. Despite this, the sample size of the exposure GWAS means that we are likely missing SNPs that could be used as instrumental variables, and so, we are unable to be definitive about our conclusion because of the lack of statistical power.

The assumptions of MR (relevance, independence, and exclusion restriction) are largely unfalsifiable. In particular, IL-6 signaling is complex, and despite the extensive literature using rs2228145 as a phenocopy of IL-6 antagonism [10,^11^,^13^,^16^,^37^,38], it is not clear how well rs2228145 performs in blocking all aspects of Il-6 signaling, and how well it can be considered to truly instrument IL-6 signaling downregulation is a matter for future research.

Our final limitation remains the challenge of interpreting germline variation relating to a lifetime exposure to changes in IL-6 signaling as evidence for or against the therapeutic usage of IL-6 antagonism in acute malaria or for elucidating the causal role of IL-6 in severe malaria. This evidence is suggestive that IL-6 is not causal and should be taken in the context that variants that alter IL-6 signaling do alter both the incidence and outcomes of other infections and that the trial evidence of IL-6 inhibition has supported the genetic evidence in COVID-19 [10,^11^,39]. However, we would caution overinterpretation of our null result to suggest that IL-6 is irrelevant in severe malaria; although, it does weaken the case for suggestion of IL-6 inhibition as a therapeutic option.

## Conclusion

Using SNPs near *IL6R* to proxy IL-6 signaling, we found no evidence that IL-6 signaling has a causal role in the development of severe malaria; although, our results had imprecision that cannot preclude a small effect. This evidence does not support the consideration of IL-6 manipulation in patients with severe malaria.

## Funding support and the role of the funding source

FH's time was funded by the GW4-CAT Wellcome Doctoral Fellowship Scheme (222894/Z/21/Z). PG's time was funded by the Ser Cymru program, the Welsh Government, and the EU-ERDF. NJT is a Wellcome Trust investigator (202802/Z/16/Z), is the princial investigator of the Avon Longitudinal Study of Parents and Children (Medical Research Council, MRC & Wellcome Trust, WT 217065/Z/19/Z), is supported by the University of Bristol National Institute for Health Research Biomedical Research Centre (BRC-1215-2001), the MRC Integrative Epidemiology Unit (MC_UU_00011/1), and works within the Cancer Research UK Integrative Cancer Epidemiology Programme (C18281/A29019). This study makes use of data generated by MalariaGEN. A full list of the investigators who contributed to the generation of the data is available from www.malariagen.net. The funding for this project was provided by Wellcome Trust (WT077383/Z/05/Z) and the Bill & Melinda Gates Foundation through the Foundation of the National Institutes of Health (566) as part of the Grand Challenges in Global Health Initiative. The funder had no role in the design, analysis, or reporting of this study.

## Ethical approval

Because this study used only publicly available data, no ethical approval was required. Details of ethical approval for the datasets used in this study are available with the original publications: UK Biobank [40], ARIC [20], and MalariaGEN [28].

## Declaration of competing interest

The authors have no competing interests to declare.

## Appendix Supplementary materials

Image, application 1

Image, application 2

## References

[1] Rose-John S (2018). Interleukin-6 family cytokines. Cold Spring Harb Perspect Biol.

[2] Rose-John S, Winthrop K, Calabrese L (2017). The role of IL-6 in host defence against infections: immunobiology and clinical implications. Nat Rev Rheumatol.

[3] Wilairatana P, Mala W, Milanez GJ (2022). Increased interleukin-6 levels associated with malaria infection and disease severity: a systematic review and meta-analysis. Sci Rep.

[4] Lyke KE, Burges R, Cissoko Y (2004). Serum levels of the proinflammatory cytokines interleukin-1 beta (IL-1beta), IL-6, IL-8, IL-10, tumor necrosis factor alpha, and IL-12(p70) in Malian children with severe Plasmodium falciparum malaria and matched uncomplicated malaria or healthy controls. Infect Immun.

[5] Mandala WL, Msefula CL, Gondwe EN (2017). Cytokine profiles in Malawian children presenting with uncomplicated malaria, severe malarial anemia, and cerebral malaria. Clin Vaccine Immunol.

[6] Wunderlich CM, Delić D, Behnke K (2012). Cutting edge: inhibition of IL-6 trans-signaling protects from malaria-induced lethality in mice. J Immunol.

[7] Grau GE, Frei K, Piguet PF (1990). Interleukin 6 production in experimental cerebral malaria: modulation by anticytokine antibodies and possible role in hypergammaglobulinemia. J Exp Med.

[8] Shankar-Hari M, Vale CL, Godolphin PJ, Fisher D, Higgins JPT, Spiga F, Savovic J, Tierney J, Baron G, Benbenishty JS, Berry LR, Broman N, Cavalcanti AB, WHO Rapid Evidence Appraisal for COVID-19 Therapies (REACT) Working Group (2021). Colman R, De Buyser SL, Derde LPG, Domingo P, Omar SF, Fernandez-Cruz A, Feuth T, Garcia F, Garcia-Vicuna R, Gonzalez-Alvaro I, Gordon AC, Haynes R, Hermine O, Horby PW, Horick NK, Kumar K, Lambrecht BN, Landray MJ, Leal L, Lederer DJ, Lorenzi E, Mariette X, Merchante N, Misnan NA, Mohan SV, Nivens MC, Oksi J, Perez-Molina JA, Pizov R, Porcher R, Postma S, Rajasuriar R, Ramanan AV, Ravaud P, Reid PD, Rutgers A, Sancho-Lopez A, Seto TB, Sivapalasingam S, Soin AS, Staplin N, Stone JH, Strohbehn GW, Sunden-Cullberg J, Torre-Cisneros J, Tsai LW, van Hoogstraten H, van Meerten T, Veiga VC, Westerweel PE, Murthy S, Diaz JV, Marshall JC, Sterne JAC. Association between administration of IL-6 antagonists and mortality among patients hospitalized for COVID-19: a meta-analysis. JAMA.

[9] Sanderson E, Glymour MM, Holmes MV (2022). Mendelian randomization. Nat Rev Methods Primers.

[10] Larsson SC, Burgess S, Gill D. (2021). Genetically proxied interleukin-6 receptor inhibition: opposing associations with COVID-19 and pneumonia. Eur Respir J.

[11] Hamilton F, Thomas M, Arnold D (2023). Therapeutic potential of IL6R blockade for the treatment of sepsis and sepsis-related death: findings from a Mendelian randomisation study. PLoS Med.

[12] Pairo-Castineira E, Clohisey S, Klaric L (2021). Genetic mechanisms of critical illness in COVID-19. Nature.

[13] Bovijn J, Lindgren CM, Holmes MV. (2020). Genetic variants mimicking therapeutic inhibition of IL-6 receptor signaling and risk of COVID-19. Lancet Rheumatol.

[14] Ferreira RC, Freitag DF, Cutler AJ (2013). Functional IL6R 358Ala allele impairs classical IL-6 receptor signaling and influences risk of diverse inflammatory diseases. PLOS Genet.

[15] Swerdlow DI, Holmes MV, Kuchenbaecker KB, Engmann JE, Shah T, Sofat R, Guo Y, Chung C, Peasey A, Pfister R, Mooijaart SP, Ireland HA, Leusink M, Langenberg C, Interleukin-6 Receptor Mendelian Randomisation Analysis (IL6R MR) Consortium (2012). Li KW, Palmen J, Howard P, Cooper JA, Drenos F, Hardy J, Nalls MA, Li YR, Lowe G, Stewart M, Bielinski SJ, Peto J, Timpson NJ, Gallacher J, Dunlop M, Houlston R, Tomlinson I, Tzoulaki I, Luan J, Boer JM, Forouhi NG, Onland-Moret NC, van der Schouw YT, Schnabel RB, Hubacek JA, Kubinova R, Baceviciene M, Tamosiunas A, Pajak A, Topor-Madry R, Malyutina S, Baldassarre D, Sennblad B, Tremoli E, de Faire U, Ferrucci L, Bandenelli S, Tanaka T, Meschia JF, Singleton A, Navis G, Mateo Leach I, Bakker SJ, Gansevoort RT, Ford I, Epstein SE, Burnett MS, Devaney JM, Jukema JW, Westendorp RG, Jan de Borst G, van der Graaf Y, de Jong PA, Mailand-van der Zee AH, Klungel OH, de Boer A, Doevendans PA, Stephens JW, Eaton CB, Robinson JG, Manson JE, Fowkes FG, Frayling TM, Price JF, Whincup PH, Morris RW, Lawlor DA, Smith GD, Ben-Shlomo Y, Redline S, Lange LA, Kumari M, Wareham NJ, Verschuren WM, Benjamin EJ, Whittaker JC, Hamsten A, Dudbridge F, Delaney JA, Wong A, Kuh D, Hardy R, Castillo BA, Connolly JJ, van der Harst P, Brunner EJ, Marmot MG, Wassel CL, Humphries SE, Talmud PJ, Kivimaki M, Asselbergs FW, Voevoda M, Bobak M, Pikhart H, Wilson JG, Hakonarson H, Reiner AP, Keating BJ, Sattar N, Hingorani AD, Casas JPThe interleukin-6 receptor as a target for prevention of coronary heart disease: a mendelian randomisation analysis. Lancet.

[16] Kappelmann N, Arloth J, Georgakis MK (2021). Dissecting the association between inflammation, metabolic dysregulation, and specific depressive symptoms: a genetic correlation and 2-sample Mendelian randomization study. JAMA Psychiatry.

[17] Georgakis MK, Malik R, Burgess S (2022). Additive effects of genetic interleukin-6 signaling downregulation and low-density lipoprotein cholesterol lowering on cardiovascular disease: a 2×2 factorial Mendelian randomization analysis. J Am Heart Assoc.

[18] Sirugo G, Williams SM, Tishkoff SA. (2019). The missing diversity in human genetic studies. Cell.

[19] Salemi R, Gattuso G, Tomasello B (2022). Co-occurrence of interleukin-6 receptor Asp358Ala variant and high plasma levels of IL-6: an evidence of IL-6 trans-signaling activation in deep vein thrombosis (DVT) patients. Biomolecules.

[20] Zhang J, Dutta D, Köttgen A (2022). Plasma proteome analyses in individuals of European and African ancestry identify cis-pQTLs and models for proteome-wide association studies. Nat Genet.

[21] Ferkingstad E, Sulem P, Atlason BA (2021). Large-scale integration of the plasma proteome with genetics and disease. Nat Genet.

[22] Reich D, Patterson N, Ramesh V (2007). Admixture mapping of an allele affecting interleukin 6 soluble receptor and interleukin 6 levels. Am J Hum Genet.

[23] Hemani G, Zheng J, Elsworth B (2018). The MR-Base platform supports systematic causal inference across the human phenome. eLife.

[24] Pan-UK Biobank. Pan-ancestry genetic analysis of the UK Biobank, https://pan.ukbb.broadinstitute.org/; 2022 [accessed 13 October 2022].

[25] Zhou W, Nielsen JB, Fritsche LG (2018). Efficiently controlling for case-control imbalance and sample relatedness in large-scale genetic association studies. Nat Genet.

[26] Willer CJ, Li Y, Abecasis GR. (2010). METAL: fast and efficient meta-analysis of genomewide association scans. Bioinformatics.

[27] Howie B, Marchini J, Stephens M. (2011). Genotype imputation with thousands of genomes. G3 (Bethesda).

[28] Malaria Genomic Epidemiology Network. Insights into malaria susceptibility using genome-wide data on 17,000 individuals from Africa, Asia and Oceania. Nat Commun 2019;10:5732. 10.1038/s41467-019-13480-z. 10.1038/s41467-019-13480-z PMC6914791 31844061

[29] World Health Organization. Management of severe malaria, https://apps.who.int/iris/bitstream/handle/10665/79317/9789241548526_eng.pdf; 2012 [accessed 05 January 2023].

[30] Skrivankova VW, Richmond RC, Woolf BAR (2021). Strengthening the reporting of observational studies in epidemiology using Mendelian randomisation (STROBE-MR): explanation and elaboration. BMJ.

[31] MalariaGEN data release. Genome-wide study of resistance to severe malaria in eleven worldwide populations: association summary statistics, https://www.malariagen.net/sppl25/; 2019 [accessed 04 November 2022].

[32] Constantinescu AE, Mitchell RE, Zheng J (2022). A framework for research into continental ancestry groups of the UK Biobank. Hum Genomics.

[33] Said S, Pazoki R, Karhunen V (2022). Genetic analysis of over half a million people characterises C-reactive protein loci. Nat Commun.

[34] Karcıoğlu Batur L, Savaş S, Girgin E (2022). Association of the IL-6R gene polymorphic variant rs2228145(C>A) with IL-6 gene polymorphisms in a healthy cohort of Turkish population. Genes Immun.

[35] Consortium GTEx (2020). The GTEx Consortium atlas of genetic regulatory effects across human tissues. Science.

[36] Wang QS, Edahiro R, Namkoong H (2022). The whole blood transcriptional regulation landscape in 465 COVID-19 infected samples from Japan COVID-19 Task Force. Nat Commun.

[37] Gill D, Georgakis MK, Walker VM (2021). Mendelian randomization for studying the effects of perturbing drug targets. Wellcome Open Res.

[38] Cupido AJ, Asselbergs FW, Natarajan P (2022). Dissecting the IL-6 pathway in cardiometabolic disease: A Mendelian randomization study on both IL6 and IL6R. Br J Clin Pharmacol.

[39] Rantala A, Lajunen T, Juvonen R (2011). Association of IL-6 and IL-6R gene polymorphisms with susceptibility to respiratory tract infections in young Finnish men. Hum Immunol.

[40] Bycroft C, Freeman C, Petkova D (2018). The UK biobank resource with deep phenotyping and genomic data. Nature.

