# Supplementary material for: The effect of interleukin-6 signaling on severe malaria: A Mendelian randomization analysis
Source: Int J Infect Dis. 2023 Apr;129:251–9. doi: 10.1016/j.ijid.2023.02.008 (PMC10728776; doi:10.1016/j.ijid.2023.02.008)

Supplementary Figures:

**Figure S1**: Raw inverse variance weighted MR estimates for each study site for the association between gp130 protein levels and severe malaria case status (via Wald Ratio). These are on the scale of an SD increase in inverse-rank normalised transformed gp130 protein levels. Note Nigeria again not shown due to imprecision.


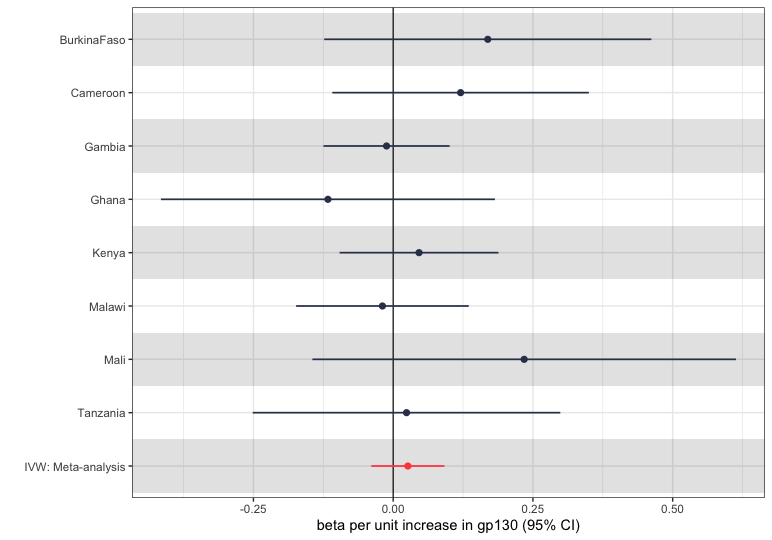


**Figure S2**: Raw inverse variance weighted MR estimates for each study site for the association between gp130 protein levels and severe malaria subtype status (via Wald ratio). These are on the scale of an SD increase in inverse-rank normalised transformed gp130 protein levels. Note Nigeria again not shown due to imprecision.


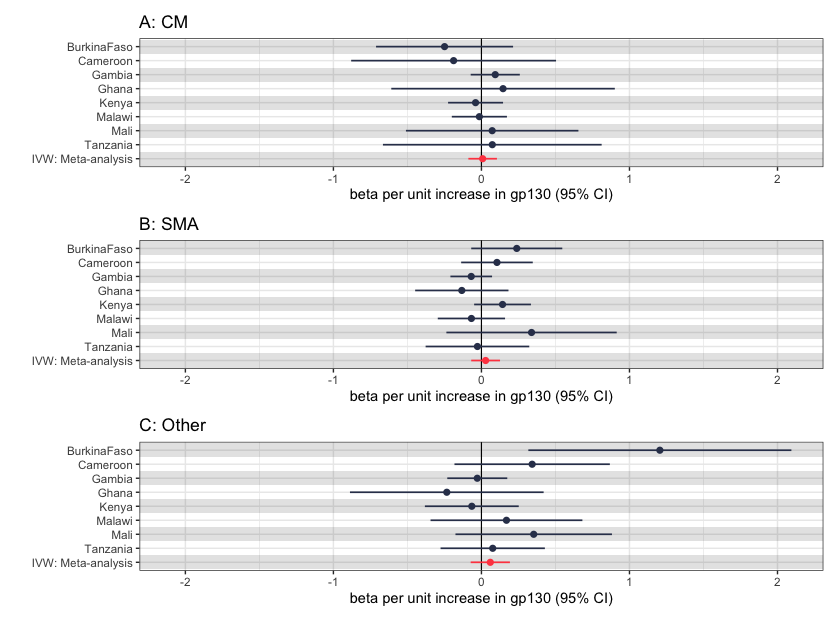

Supplement: Supplementary file 1 [file mmc1.docx]
